# Development of polyvinyl alcohol nanofiber scaffolds loaded with flaxseed extract for bone regeneration: phytochemicals, cell proliferation, adhesion, and osteogenic gene expression

**DOI:** 10.3389/fchem.2024.1417407

**Published:** 2024-07-31

**Authors:** Ahmed G. Abdelaziz, Hassan Nageh, Mohga S. Abdalla, Sara M. Abdo, Asmaa A. Amer, Samah A. Loutfy, Nasra F. Abdel Fattah, Ali Alsalme, David Cornu, Mikhael Bechelany, Ahmed Barhoum

**Affiliations:** ^1^ Biochemistry Division, Chemistry Department, Faculty of Science, Helwan University, Cairo, Egypt; ^2^ Nanotechnology Research Centre (NTRC), The British University in Egypt, Cairo, Egypt; ^3^ Department of Pharmacognosy, Pharmaceutical and Drug Industries Research Institute, National Research Centre, Cairo, Egypt; ^4^ Virology and Immunology Unit, Cancer Biology Department, National Cancer Institute, Cairo University, Cairo, Egypt; ^5^ Department of Chemistry, College of Science, King Saud University, Riyadh, Saudi Arabia; ^6^ Institut Européen des Membranes (IEM), UMR 5635, University of Montpellier, ENSCM, CNRS, Montpellier, France; ^7^ Gulf University for Science and Technology, GUST, Mubarak Al-Abdullah, Kuwait; ^8^ NanoStruc Research Group, Chemistry Department, Faculty of Science, Helwan University, Cairo, Egypt

**Keywords:** flaxseed extract, bone tissue engineering, electrospinning, nanofibrous scaffold, cell proliferation, osteoblast differentiation, regenerative medicine, MTT

## Abstract

**Introduction:** Bone tissue engineering seeks innovative materials that support cell growth and regeneration. Electrospun nanofibers, with their high surface area and tunable properties, serve as promising scaffolds. This study explores the incorporation of flaxseed extract, rich in polyphenolic compounds, into polyvinyl alcohol (PVA) nanofibers to improve their application in bone tissue engineering.

**Methods:** High-performance liquid chromatography (HPLC) identified ten key compounds in flaxseed extract, including polyphenolic acids and flavonoids. PVA nanofibers were fabricated with 30 wt.% flaxseed extract (P70/E30) via electrospinning. We optimized characteristics like diameter, hydrophilicity, swelling behavior, and hydrolytic degradation. MG-63 osteoblast cultures were used to assess scaffold efficacy through cell adhesion, proliferation, viability (MTT assay), and differentiation. RT-qPCR measured expression of osteogenic genes *RUNX2*, *COL1A1*, and *OCN*.

**Results:** Flaxseed extract increased nanofiber diameter from 252 nm (pure PVA) to 435 nm (P70/E30). P70/E30 nanofibers showed higher cell viability (102.6% vs. 74.5% for pure PVA), although adhesion decreased (151 vs. 206 cells/section). Notably, P70/E30 enhanced osteoblast differentiation, significantly upregulating *RUNX2*, *COL1A1*, and *OCN* genes.

**Discussion:** Flaxseed extract incorporation into PVA nanofibers enhances bone tissue engineering by boosting osteoblast proliferation and differentiation, despite reduced adhesion. These properties suggest P70/E30’s potential for regenerative medicine, emphasizing scaffold optimization for biomedical applications.

## Graphical Abstract

**Figure:**
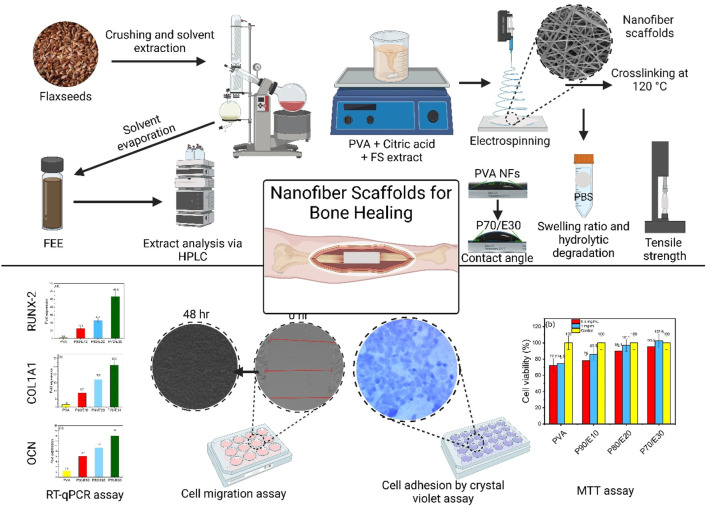
Tailored PVA nanofiber scaffolds, loaded with a flaxseed extract and optimized for bone engineering, promote MG-63 osteoblast functionalities and significantly upregulate osteoblast-like cells gene expression, showcasing their strong potential for bone tissue regeneration.

## Highlights

• PVA nanofiber scaffolds loaded with a flaxseed extract were designed for bone tissue engineering.
• Nanofiber scaffolds with different flaxseed extract concentrations were characterized using different techniques.
• Nanofiber scaffolds promoted osteoblast proliferation and differentiation with reduced adhesion.
• The significant MG-63 osteoblast-like cells gene expression upregulation showed the scaffold potential for supporting bone regeneration.

## 1 Introduction

Bone, a mineralized connective tissue, provides support to soft tissues, enables movement, houses bone marrow, and is the main reservoir of calcium and phosphate in the form of hydroxyapatite crystals (Lin et al., 2020). Bone tissue damage, caused by pathological conditions or traumatic injuries, results in over 2.2 million bone graft procedures per year worldwide. Presently, bone damage is managed using metal/ceramic prostheses, allogenic, and autologous grafts (Wickramasinghe et al., 2022). However, graft-based approaches in bone damage treatment present multiple drawbacks, including the need for multiple surgical interventions, donor site pain/discomfort, inadequate graft supply for extensive segmental bone loss, and high risk of immune rejection of non-autologous grafts (Abdelaziz et al., 2023; Selim et al., 2024). The current challenges and limitations of the existing bone damage treatments underscore the urgent need for alternative bone tissue regeneration approaches. Plant-derived extracts represent a promising avenue to address these shortcomings. Indeed, the incorporation of plant extracts into polymeric scaffold materials offers a potential solution to enhance bone healing and regeneration, while mitigating the shortcomings associated with conventional graft-based approaches. The plant extracts bioactive properties might stimulate osteogenesis by promoting cell adhesion, proliferation, and differentiation, which are crucial for effective bone tissue regeneration (Djehiche et al., 2024a).

In bone tissue engineering, polymeric scaffolds sourced from natural (chitosan, cellulose, starch) or synthetic polymers (polyethylene glycol, poly (ε-caprolactone), polyvinylpyrrolidone) display specific and unique advantages and challenges (Nasrin et al., 2017; Leung et al., 2022). Natural polymers mimic the native extracellular matrix to support a biomimetic environment with reduced immune response, biodegradability, and multiple adhesion sites (Nasrin et al., 2017; Leung et al., 2022). However, their drawbacks include lower mechanical strength, cross-contamination, and low thermal stability (Bhatia, 2016; Salehi-Nik et al., 2017). Conversely, synthetic polymers offer extended shelf life, higher mechanical properties, purity, and efficient mass production. Yet, they pose challenges, such as limited bioactivity and potential long-term effects, especially non-degradable polymers, and the formation of cytotoxic degradation byproducts (Hartley et al., 2022; Terzopoulou et al., 2022). Polymeric nanofiber scaffolds, a recent advancement (Barhoum et al., 2019), could be used for bone tissue engineering and regeneration (Rasouli et al., 2019). However, the precise control of the nanofiber properties (morphology, fiber diameter, pore size, surface chemistry, and wettability) is crucial for optimizing their efficacy in bone tissue engineering (Barhoum et al., 2018; Gugulothu et al., 2019). Tailoring these characteristics allows optimized microenvironment, which is essential for cell interaction, growth, and differentiation and consequently for successful bone tissue regeneration (Zhao et al., 2022).

Flax (*Linum usitatissimum* L.) seeds boast a long medicinal history attributed to their high omega-3 fatty acid content (Pereira et al., 2022). Several studies have reported that flaxseed extract contains a diverse array of polyphenolic compounds and flavonoids (Noreen et al., 2023; Koçak, 2024; Wang et al., 2024). These bioactive molecules have been shown to promote osteogenesis through various molecular signaling pathways, including the estrogen receptor (ER) signalling pathway (Cauley, 2015), mitogen-activated protein kinases (MAPK) (Torre, 2017), Wnt/β-catenin (Torre, 2017), and tumour growth factor-beta/bone morphogenetic protein (TGF-β/BMP) pathways (Melguizo-Rodríguez et al., 2019). This study investigated the integration of a flaxseed extract into polyvinyl alcohol (PVA) nanofibrous scaffolds for bone tissue engineering. The primary aim was to evaluate how the extract incorporation influenced the regeneration of damaged bone tissue. Our findings emphasize the pivotal roles played by the nanofiber characteristics (diameter, morphology, pore size, surface chemistry, and wettability) in promoting cell adhesion, proliferation, and differentiation, which are crucial for bone tissue regeneration. Various techniques, including Fourier transform infrared (FTIR) spectroscopy, field emission scanning electron microscopy (FE-SEM), water contact angle (WCA) measurements and mechanical tests, were employed to analyse the nanofibrous scaffold morphology and properties. Reverse transcription-quantitative polymerase chain reaction (RT-qPCR) analysis was used to monitor the expression of genes linked to osteoblast differentiation. Despite the promising results, one of the major challenges will be to optimize the extract loading ratio for maximum bioactivity without compromising the scaffold integrity, while ensuring overall biocompatibility and safety. Addressing these challenges is imperative for unlocking the full potential of plant extracts to develop innovative nanofibrous scaffolds for bone tissue engineering and regeneration.

## 2 Experimental

### 2.1 Flaxseeds extract preparation

The dried flaxseeds *L. usitatissimum* L. were purchased from a local farm in Shubra Mills, Gharbia Governorate, and identified by a professor of taxonomy, faculty of science, Cairo University. The flaxseeds were washed with deionized milli-Q water, and then the seeds were crushed. One hundred grams of crushed seeds were immersed in 2,000 mL of boiled 70% ethyl alcohol (C_2_H_5_OH, 99% purity, Piochem, Egypt) for the extraction of bioactive compounds. This process was repeated five times for complete extraction of phytochemical constituents. The resulting ethanol extract was concentrated using a rotary evaporator at 52°C until dryness, yielding a viscous, brownish-coloured extract stored at −20°C. Quantitative estimation and identification of the extract components were performed using high-performance liquid chromatography (HPLC) (Wollenweber, 1983; Djehiche et al., 2024b), as precisely described in the Supplementary Material.

### 2.2 Nanofibrous scaffold fabrication and characterization

The electrospinning process involved preparing solutions with different dry weight ratios of PVA (molecular weight of 85,000–124,000 g/mol, 87%–89% hydrolyzed, Sigma Aldrich, Germany) and of total ethanolic flaxseed extract. Each 15 mL solution, containing 1.5 g of PVA and extract, yielded a final concentration of 10% (w/v). Four solutions were prepared: i) PVA90/E10 (PVA: extract ratio = 90:10), ii) P80/E20 (PVA: extract ratio = 80:20), iii) P70/E30 (PVA: extract ratio = 70:30), and iv) PVA (8.5% of PVA) (Supplementary Table S1). PVA solutions were prepared by dissolving PVA in 10 mL deionized milli-Q water, stirring at 85°C overnight, followed by refrigeration. Extract solutions were made by dissolving the extract in 5 mL deionized milli-Q water and stirring at 65°C for 3 h. The PVA/extract solutions were combined, and 0.225 g of 1.5% (w/v) citric acid (C_6_H_8_O_7_, Fisher Bioreagents, Massachusetts, United States) was added for homogeneity. After sonication to remove air bubbles, each solution was electrospun using an NANON-01A electrospinner (MECC, Japan) (operational conditions in Supplementary Table S1), in ambient conditions (35%–46% humidity), followed by vacuum drying at 50°C. For stability in the aqueous-based media used in*in vitro* studies, nanofibers were crosslinked at 120°C for 2 h and were stored at −20°C. The fabricated nanofiber characteristics are detailed in Supplementary Table S2.

Electrospun nanofibers were investigated using FTIR (Brucker Vertex 70, United States), FE-SEM (ThermoFisher, Quattro S FEG ESEM, United States), WCA measurements (Kruss, drop shape analyzer DSA25B, Germany), and tensile strength (TA Instruments, Dynamic Mechanical Analyzer Q800, United States). FE-SEM images were analyzed with the Image J software (version 1.53t) to determine the mean nanofiber diameter, and histograms were constructed using OriginLab 2018. The nanofiber physicochemical analysis included assessing their hydrolytic degradation following the method outlined by Hussein et al. (2020). Nanofiber discs were cut into 6 mm discs and after recording their initial weight, they were submerged in 0.1 M phosphate buffer saline (PBS, Gibco, South America) pH = 7.4. Then, weight was recorded, after complete drying, every 24 h for 7 days to document weight loss. The hydrolytic degradation rate was calculated using the following formula Eq. (1):

$$\text{Hydrolytic degradation }\%=\frac{Wi-Wd}{Wi}\times 100$$ (1)

(Hussein et al., 2020). where W_i_ and W_d_ are the initial weight and the weight of dried nanofibers at time T, respectively.

### 2.3 Cell culture and cytotoxicity analysis (MTT assay)

The human osteosarcoma osteoblast-like MG-63 cell line was provided by Nawah Scientific, Cairo, Egypt. Cells were maintained in Dulbecco’s Modified Eagle Medium (DMEM, Lonza, NJ, United States) with 10% fetal bovine serum (Gibco, South of America), 10 mg/mL streptomycin (Lonza, NJ, United States), 2 v/v% penicillin (Lonza, NJ, United States), 1 v/v% sodium pyruvate (Lonza, NJ, United States), and 1 v/v% L-glutamine (Lonza, NJ, United States) in standard conditions (37°C, 5% CO_2_ and 95% humidity). Cells were passaged using trypsin solution (0.025 w/v% ethylenediaminetetraacetic acid and 0.025 w/v% trypsin) followed by washing in PBS (Gibco, South America) to remove cell debris. The cytotoxicity of the flaxseed extract, PVA nanofibers, and PVA/extract nanofibers was assessed with the MMT assay according to Mosmann (1983). Various concentrations of extract (400, 200, 100, 50, and 25 μg/mL) and PVA/extract nanofibers (0.5 and 1 mg/mL) were tested. MG-63 cells (104 cells/well) were cultured in 96-well plates for 24 h. After reaching >90% of confluency, medium was replaced with fresh medium containing the tested materials (extract and scaffolds) at different concentrations. After incubation in the dark for 48 h, 2% MTT solution was added, and plates were incubated for 4 h. After discarding the MTT solution, DMSO was added to dissolve the formazan crystals for 15 min. Absorbance was determined by measuring the optical density at 570 nm using a microplate reader (CLARIOstar^®^ Plus, BMG Labtech, Ortenberg, Germany). Cell viability was calculated using the following formula Eq. (2):

$$\text{Relative cell viability }\left(\%\right)=\left[A_{s}/A_{c}\right]\times 100$$ (2)

(Mosmann, 1983). where A_s_ and A_c_ are the mean absorbance values of the test sample and control sample, respectively.

### 2.4 Cell migration assay on MG-63 cell line

MG-63 cell migration was assessed according to Hadisi et al. (2020). Briefly, 1 × 10^5^ MG-63 cells/well were cultured in 12-well plates. After reaching 85%–90% of confluency, a controlled gap was made in the cell layer using a 200 µL pipette tip in a North-to-South direction. After rinsing with PBS to remove debris, nanofiber scaffolds (0.5 and 1 mg/mL) were introduced in each well and cells were kept in standard conditions. The gap closure (%) was evaluated at 0, 24, and 48 h post-scratching using an inverted fluorescence microscope (Axio Observer 5, Carl Zeiss, Germany). Images were captured with a digital camera (Axiocam 512 Color). The gap area was measured using the ZEN2.3 image analysis software (Carl Zeiss, Germany), and the percentage of gap closure was calculated using the following formula Eq. (3):

$$\text{Gap closure }\left(\%\right)=\frac{\text{Gap}\;\text{area}\;\text{at}\;0\;h-\text{Gap}\;\text{area}\;\text{at}\;x\;h}{\text{Gap}\;\text{area}\;\text{at}\;0\;h}\times 100$$ (3)

(Hadisi et al., 2020).

### 2.5 Cell adhesion assay on MG-63 cell line using crystal violet

Cell adhesion on nanofibrous scaffolds was evaluated using crystal violet staining following the procedure described by Wu et al. (2019) with some modifications. Nanofiber scaffolds (6 mm diameter discs) were sterilized in 70% ethanol/PBS, placed in a 24-well plate and soaked in 500 µL of PBS for 2 h. After removing the excess PBS, 250 µL of MG-63 cell suspension (1 × 10^5^ cells/mL in DMEM) was seeded onto the nanofiber scaffold and incubated at room temperature for 3 h to facilitate cell adhesion. Non-adherent cells were gently rinsed off twice with PBS. Following adhesion, cells were fixed with 500 µL of 10% formaldehyde in PBS for 15 min, then rinsed twice with 500 µL of PBS. For staining, 200 µL of 0.25% crystal violet dye (SERVA Electrophoresis GmbH, Germany) in 20% ethanol was added to each well for 30 min. After rinsing with PBS to remove the excess crystal violet, images were acquired with an Inverted Fluorescence Microscope (Axio Observer 5, Carl Zeiss, Germany) and a ×20 objective, and cell were counted in each image.

### 2.6 Osteogenic differentiation of MG-63 by quantitative real time polymerase chain reaction assay

The nanofiber scaffold osteogenic potential was assessed by quantifying the expression of three key osteogenesis-related genes: runt-related transcription factor 2 variant 1 (*RUNX2* V1), osteocalcin (*OCN*), and collagen type I (*COL1A1*) by RT-qPCR. Primers for each gene were designed with the NCBI primer designing tool primer blast, and beta-actin (*ACTB*) primers were from Loutfy et al (2017) (Table 1). MG-63 cells (400,000 cells/well) were plated in six-well plates with nanofibers (1 mg/mL) for 72 h. After harvesting, total mRNA was isolated using the GeneJET RNA Purification Kit (Thermo Scientific, Inc., United States) and reverse transcribed to complementary DNA (cDNA) using oligodT primers and the RevertAid RT Reverse Transcription Kit (Thermo Scientific, United States). The qPCR mix included 12.5 μL SYBR Green reagent, 3 μL of each specific primer and internal control primers, 2 μL cDNA, and 4.5 μL molecular-grade water in a total volume of 25 μL. The *ACTB* housekeeping gene was used as a control.

**TABLE 1:** Forward and reverse PCR primers for the indicated genes.

Gene	Primer
*RUNX-2*	Forward primer 5′- AGCCACCGAGACCAACAGAG-3′ Reverse primer 5′- GTGTCACTGTGCTGAAGAGGC-3′
*OCN*	Forward primer 5′-CAGCCACCGAGACACCATGAG-3′ Reverse primer 5′-TTGATACAGGTAGCGCCTGGG-3′
*COL1A1*	Forward primer 5′- GCTCGTGGAAATGATGGTGC-3′ Reverse primer 5′- ACACCCTGGGGACCTTCAGA-3′
*ACTB*	Forward primer 5′- CTGTCTGGCGGCACCACC-3′ Reverse primer 5′- GCAACTAAGTCATAGTCCGC-3′

## 3 Results and discussion

### 3.1 Phytochemical analysis

HPLC analysis of the total ethanol extract of flaxseeds identified ten compounds, including four phenolic acids and six flavonoids. Key flavonoids such as apigenin, kaempferol, quercetin, hesperidin, catechin, and rutin, along with prominent phenolic acids like chlorogenic acid, caffeic acid, and gallic acid, were detected. Chlorogenic acid exhibited the highest concentration (41.81%) as a major phenolic acid, and apigenin (16.88%) and rutin (14.87%) as major flavonoid contents. These compounds are known for their potent antioxidant properties, essential for scavenging reactive oxygen species (ROS) (Hassanpour and Doroudi, 2023). The detailed quantitative data in Table 2, including the retention time and concentration percentage for each compound, provides valuable insights into the relative abundance of these bioactive constituents in the flaxseed extract.

**TABLE 2:** HPLC analysis of the total ethanol extract of flaxseeds.

Identified compound	Concentration (%)	Retention time (min)
Gallic acid	7.26	9.550
Catechin	2.67	22.832
Caffeic acid	9.29	26.951
Chlorogenic acid	41.81	29.448
Ellagic acid	1.10	42.491
Rutin	14.87	43.181
Hesperidin	1.53	45.103
Quercetin	0.88	53.521
Kaempferol	3.70	57.095
Apigenin	16.88	57.733

The identified compounds, including gallic acid, catechin, caffeic acid, chlorogenic acid, ellagic acid, rutin, hesperidin, quercetin, kaempferol, and apigenin, each exhibit distinct antioxidant and anti-inflammatory properties. Integrated into scaffolds, they hold potential for enhancing bone healing processes. Gallic acid, with its antioxidant and anti-inflammatory properties, has shown promise in mitigating oxidative stress and inflammation associated with bone disorders, thus aiding in bone tissue regeneration (Zhang et al., 2021). In a study by Li G. et al. (2023), the incorporation of gallic acid into an injectable gelatin-based hydrogel demonstrated encouraging results in treating osteoarthritis. Similarly, catechin’s antioxidant and anti-inflammatory effects contribute to bone health by reducing oxidative damage and inflammation (Sugawara et al., 2024). Caffeic acid also promotes bone healing by mitigating oxidative stress and inflammation within the bone microenvironment (Chen et al., 2024). Research by Kızıldağ et al. (2020), revealed that administration of caffeic acid phenethyl ester (CAPE) significantly reduced oxidative stress and bone loss in diabetic rats after 15 days of administration.

Chlorogenic acid, renowned for its antioxidant properties, holds potential in bone tissue engineering by scavenging free radicals and shielding against oxidative damage, thereby bolstering bone health (Rogozinska et al., 2023). In a recent study by Nishida et al. (2023), chlorogenic acid demonstrated protective effects against alveolar bone loss in a ligature-induced periodontitis rat model, attributed to its anti-inflammatory properties. Ellagic acid, known for its antioxidant and anti-inflammatory effects, could facilitate bone tissue regeneration by diminishing oxidative stress and inflammation (Baradaran Rahimi et al., 2020). In a recent study by Dong et al. (2024), ellagic acid promoted osteogenic differentiation *in vitro* on mesenchymal stem cells (MSCs) by increasing the expression of several osteogenic genes, including RUNX-2, Osterix, ALP, COL1A1, osteopontin, and osteocalcin. Furthermore, oral supplementation with ellagic acid led to an increase in bone mass in ovariectomized mice during the study period.

Rutin possesses antioxidant and anti-inflammatory properties, potentially supporting bone health by mitigating oxidative damage and inflammation in the bone tissue (Tobar-Delgado et al., 2023). In a recent study conducted via Albaqami et al (2023), rutin gel was incorporated into bone allografts to treat induced bone defects in New Zealand rabbits and the authors reported significant enhancement in bone healing and inhibition in bone metalloproteases and increase in collagen III production. Hesperidin displays antioxidant and anti-inflammatory activities, which may contribute to bone tissue engineering by reducing oxidative stress and inflammation, thus promoting bone healing (Buzdağlı et al., 2023). In recent research conducted via Miguez et al (2024), hesperidin was supplemented with BMP to Sprague–Dawley rats with critical-size mandible defects, the authors reported that rats treated with hesperidin and BMP had a significant decrease in inflammatory markers and fully fused mandibles compared to rats treated with BMP only.

Quercetin exhibits antioxidant, and anti-inflammatory properties which could support bone health by reducing oxidative stress, and inflammation (Al-Ansari et al., 2023). In a recent study conducted via Yurteri et al (2023), quercetin was supplemented to rats with induced fractures. According to histopathological and biochemical investigations, it was observed that quercetin-treated rats had significantly higher bone healing rate compared to control groups. Kaempferol displays antioxidant and anti-inflammatory effects, which may contribute to bone tissue engineering by mitigating oxidative stress and inflammation, supporting bone healing processes (Luis Cansian et al., 2023). Kaempferol efficacy in enhancing bone healing was shown in a recent study at which it was incorporated in bioactive glass scaffolds. The study indicates that the incorporation of Kaempferol significantly enhanced bone healing rates and quality. This improvement was confirmed through immunohistochemical, histomorphometric, radiological, and three-dimensional micro-computed tomography analyses (Ranjbar et al., 2023). Apigenin exhibits antioxidant, anti-inflammatory, and potential anti-cancer properties, which could aid in bone tissue regeneration by reducing oxidative stress, inflammation, and potentially inhibiting bone cancer growth, thereby promoting bone health (Li Z. et al., 2023). Apigenin-loaded chitosan/gelatin membrane was reportedly developed to be utilized in bone tissue engineering applications, the study revealed Saos-2 osteoblast cells cultured on the fabricated apigenin-loaded membranes had higher calcium depositions, proliferation and viability compared to chitosan/gelatin membranes (Bozorgi et al., 2023).

### 3.2 Morphological analysis of the nanofiber scaffolds

**FIGURE 1:**
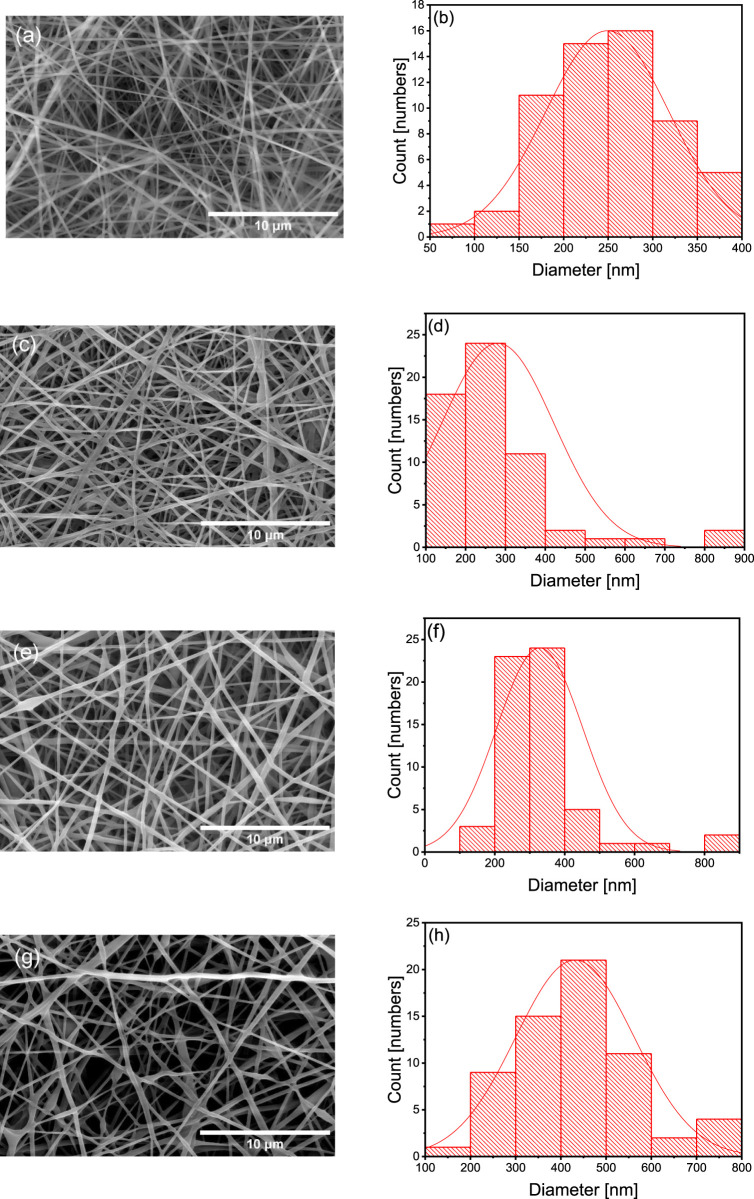
SEM images at 8,000× magnification **(A,C,E and G)** alongside the corresponding histograms displaying the diameter distribution **(B,D,F and H)** of various nanofibers **(A and B)** PVA nanofibers, **(C and D)** P90/E10 nanofibers, **(E and F)** P80/E20 nanofibers, and **(G and H)** P70/E30 nanofibers.

Figure 1 presents SEM images and the diameter distribution histograms of the different nanofiber scaffolds. The diameter increased with the flaxseed extract concentration (PVA = 252 ± 62 nm, P90/E10 = 276 ± 124 nm, P80/E20 = 326 ± 104 nm, and P70/E30 = 435 ± 119 nm), suggesting a proportional effect of the flaxseed extract. Uniform, droplet-free nanofiber scaffolds were obtained with the P90/E10 and P80/E20 formulations. The water and citric acid contents (13.5 mL and 0.225 g, respectively) are in line with previous findings by Woo and Lee (2021). These results indicated that the extract solution viscosity increased with the polyphenol-rich extract content, resulting in larger nanofiber diameters. When PVA solutions are electrospun, citric acid and polyphenols act as crosslinking agents through distinct mechanisms. Citric acid (with its small size and well-defined carboxylic acid groups) forms ester bonds via a chemical reaction, leading to robust and covalent crosslinking with PVA hydroxyl groups. Polyphenols from plant extract (with multiple phenolic hydroxyl groups) contribute to crosslinking through physical interactions like hydrogen bonding, providing a weaker form of crosslinking.

### 3.3 Tensile strength and elongation at break

**FIGURE 2:**
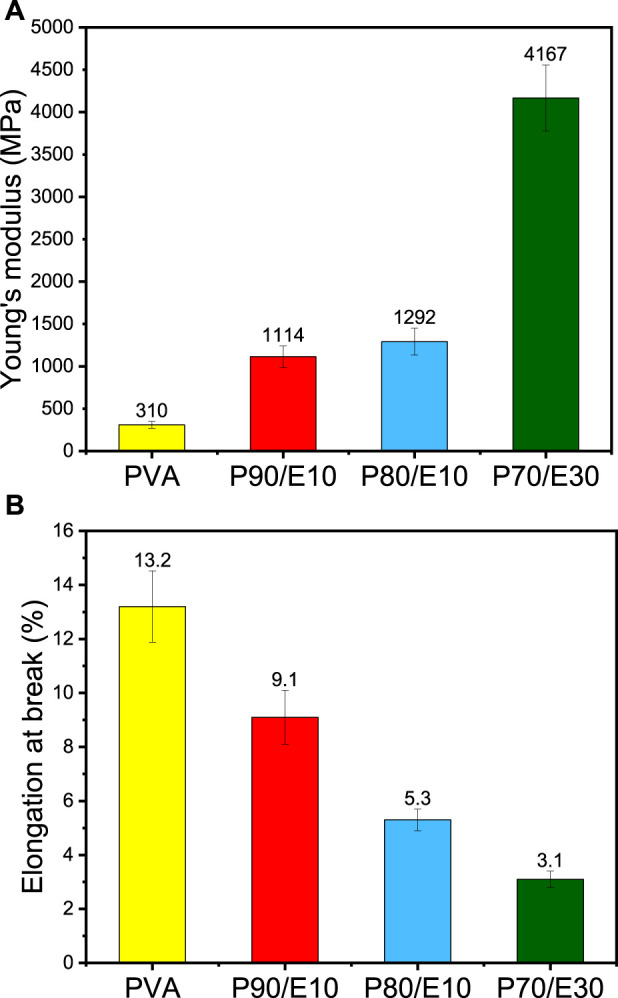
Mechanical properties of PVA nanofiber scaffolds loaded or not with flaxseed extract (different concentrations): **(A)** Young’s modulus, **(B)** elongation at break.

The effect on Young’s modulus and elongation at the break of adding the flaxseed extract (different concentrations) in PVA nanofibers are summarized in Figure 2 and Supplementary Table S3. The Young’s modulus increased from 310 ± 41 MPa for PVA to 4167 ± 391 MPa for P70/E30 (Figure 2A), indicating improved mechanical integrity with higher extract concentrations. Conversely, elongation at break decreased with higher extract concentrations (Figure 2B), from 13.0% ± 1.32% for PVA to 3.0% ± 0.28% for P70/E30, indicating reduced material flexibility. Similarly, Luo et al. (2020) showed that tea polyphenols with PVA increased the nanofiber diameter and mechanical properties. Flaxseed extract inclusion in PVA nanofiber scaffolds enhanced the mechanical properties. Indeed, the synergistic effect of robust chemical crosslinking by citric acid and weak physical crosslinking by polyphenols enhanced the overall structural stability of the nanofibers, resulting in improved mechanical properties (e.g., tensile strength and flexibility). Larger nanofiber diameters, obtained with higher extract concentrations, play a crucial role in increasing the scaffold’s mechanical strength, thus making it more suitable for bone tissue engineering applications.

### 3.4 Water contact angle, swelling, and hydrolytic degradation

**FIGURE 3:**
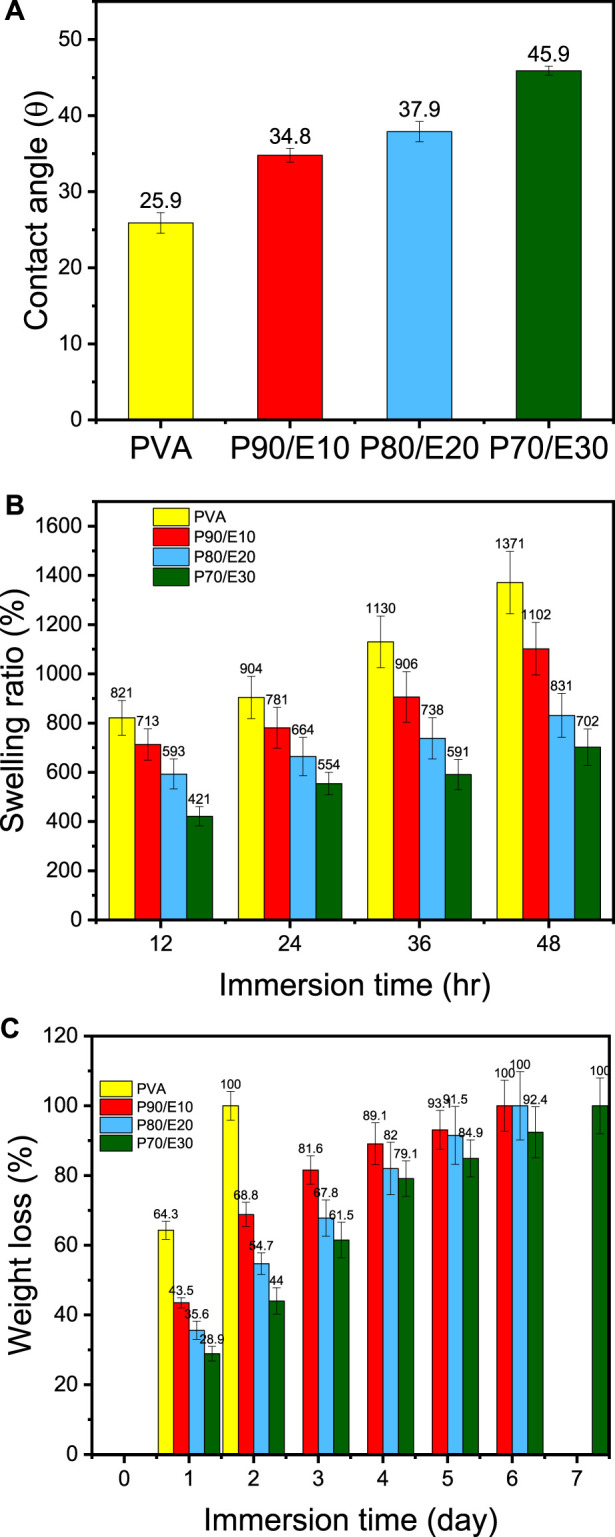
Physicochemical characterization of PVA nanofiber scaffolds loaded or not with flaxseed extract: **(A)** Water contact angle, **(B)** Swelling ratio percentage, and **(C)** Weight loss percentage after immersion in water for the indicated time.

The WCA, a surface wettability measure, increased with the flaxseed extract concentration: PVA (26.0°), P90/E10 (34.8°), P80/E20 (38.0°), and P70/E30 (45.9°) (Figure 3A). This can be explained by the presence in the flaxseed extract of amphipathic polyphenolic compounds that increase the WCA, thus decreasing surface wettability. The swelling ratio is a crucial measure of the water absorption capacity of nanofiber scaffolds for biological applications. This ratio decreased proportionally with the flaxseed extract content: PVA (1371%), P90/E10 (1102%), P80/E20 (831%), and P70/E30 (702%) (Figure 3B). This suggests that flaxseed extract enhances the nanofiber scaffold’s hydrophilic nature, fostering interactions with biological fluids. The hydrolytic degradation data (Figure 3C) confirmed the impact of extract loading: PVA (100% of weight loss after 2 days), P90/E10 (100% of weight loss after 6 days), P80/E20 (100% of weight loss after 6 days), and P70/E30 (100% of weight loss after 7 days) (for detailed hydrolytic degradation and swelling ratio results see Supplementary Table S4). The longer time needed for complete hydrolytic degradation of scaffolds with higher extract concentrations, especially P70/E30, indicates improved stability attributed to the robust crosslinking between the extract polyphenols and PVA via citric acid. Previous studies also showed increased aqueous stability after plant extract incorporation in PVA nanofibers (Quintero-Borregales et al., 2023). Incorporating flaxseed extract into PVA nanofibrous scaffolds significantly changed their water-related properties, including WCA, swelling behavior, and hydrolytic degradation, highlighting the complex interplay between flaxseed extract and PVA, relevant for tissue engineering applications.

### 3.5 Cell viability via MTT assay

**FIGURE 4:**
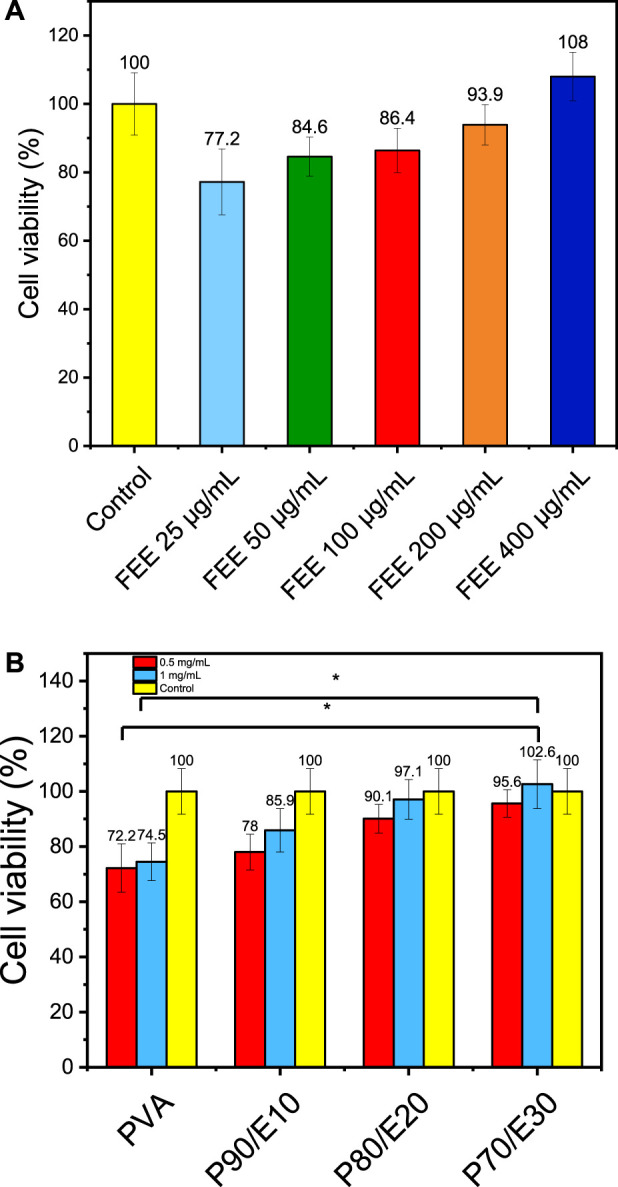
MTT assay to evaluate MG-63 osteoblast-like cell viability in the presence or not (control with medium alone) of **(A)** flaxseed ethanolic extract (FEE) and **(B)** the different nanofiber scaffolds at the indicated concentrations. One asterisk (*) indicates *p*-value < 0.05, two asterisks (**) indicate *p*-value < 0.01, and three asterisks (***) indicate *p*-value < 0.001 (compared to both control and PVA NFs results respectively).

*Cell viability* assay data were statistically analyzed using a one-way analysis of variance (ANOVA) test, performed with SPSS 19 software. The effect of different concentrations of flaxseed extract (25, 50, 100, 200, and 400 μg/mL) and nanofiber scaffolds (0.5 and 1 mg/mL) on MG-63 cell viability was assessed with the MTT assay (Figure 4 and Supplementary Table S5). Compared with control (medium alone), cell viability was slightly decreased in the presence of the extract/scaffolds. However, in cultures with the extract/scaffolds, cell viability increased with higher concentrations of flaxseed extract and extract-loaded nanofiber scaffolds. The highest viability (108.0% ± 7.1% vs. control) was observed with 400 μg/mL of flaxseed extract. Lower extract concentrations (25–200 μg/mL) only slightly affected cell viability compared with control. In the presence of 0.5 mg/mL nanofiber scaffolds, cell viability ranged from 72.2% ± 8.8% with PVA to 78.5% ± 6.5% with P90/E10, 90.1% ± 5.2% with P80/E20, and 95.6% ± 5.0% with P70/E30. Similar results were observed with a 1 mg/mL scaffold (from 74.5% ± 6.8% to 102.6% ± 8.8%). P70/E30 cell viability at a concentration of 1 mg/mL was significantly higher (*p*-value < 0.05) than PVA NFs at a concentration of 0.5 and 1 mg/mL. Flaxseed extract incorporation positively influenced the cell metabolic activity, creating a biocompatible environment for cell proliferation. This is attributed to the cell proliferation-promoting effects of the phenols present in the extract, known to downregulate inflammatory mediators, thus emphasizing the scaffold cytocompatibility, crucial for bone tissue engineering. The changes in the water-related properties (WCA and swelling ratio) might facilitate interactions with biological fluids, supporting nutrient exchange. The improved nanofibers stability (longer time to complete hydrolytic degradation) also contributes to a sustained environment for cell growth, and the larger nanofiber diameters reinforce the scaffold mechanical strength, providing a suitable substrate for cell adhesion and proliferation.

### 3.6 Cell migration on MG-63 cell line

**FIGURE 5:**
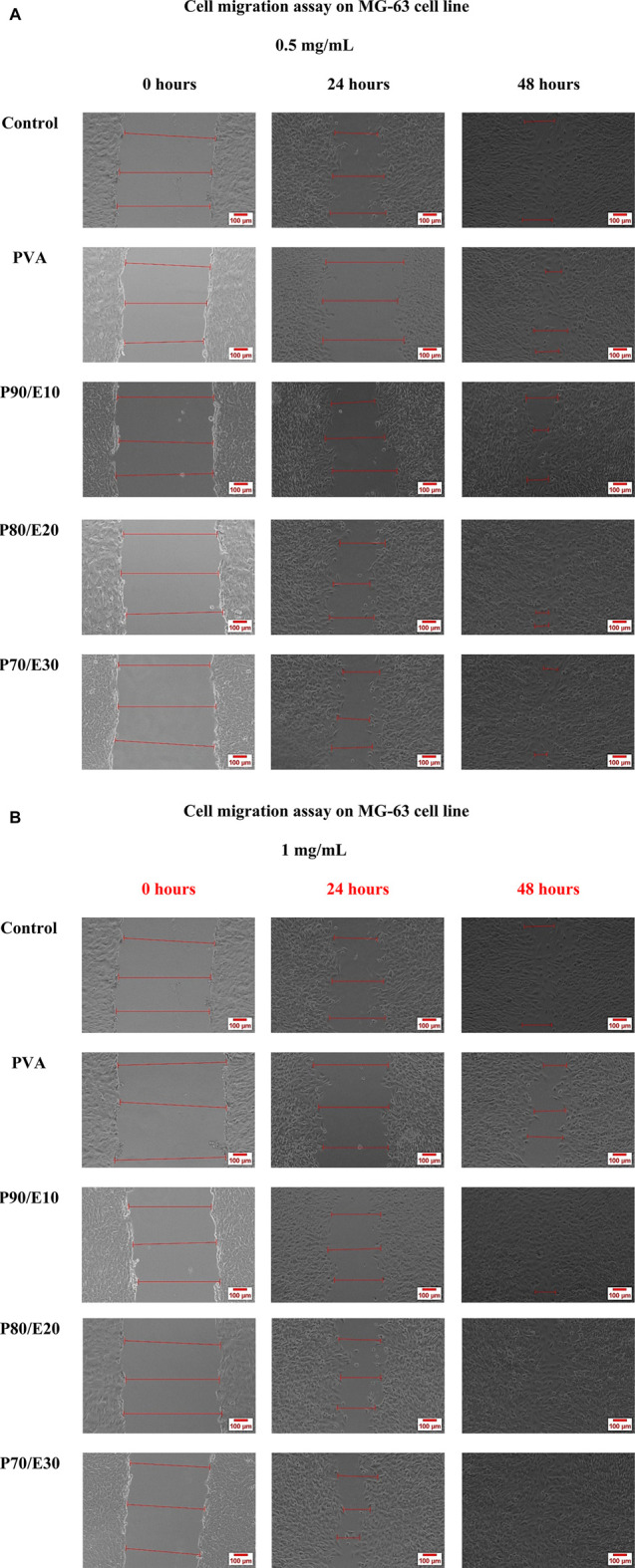
MG-63 cell migration assay in the presence or not (control, medium only) of the indicated nanofiber scaffolds at the concentration of **(A)** 0.5 mg/mL and **(B)** 1 mg/mL.

**FIGURE 6:**
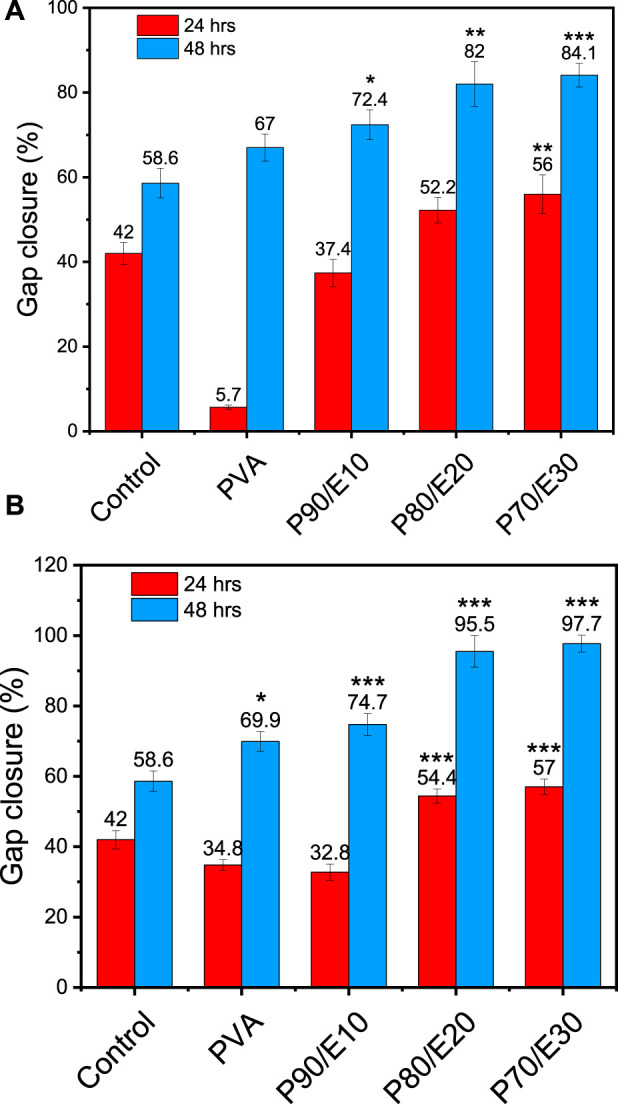
Gap closure (%) in MG-63 cell cultures in the presence or not (control, medium alone) of the indicated PVA nanofiber scaffolds with and without flaxseed extract at **(A)** 0.5 mg/mL, and **(B)** 1 mg/mL at 24 and 48 h post-scratching One asterisk (*) indicates *p*-value < 0.05, two asterisks (**) indicate *p*-value < 0.01, and three asterisks (***) indicate *p*-value < 0.001 (compared to control results at 24 and 48 h).

The gap closure percentage was estimated at 24- and 48-h post-scratching to obtain insights into the MG-63 cell migratory behavior in the presence of PVA nanofiber scaffolds with and without flaxseed extract (0.5 and 1 mg/mL) (Figures 5, 6 and Supplementary Table S6). When 0.5 mg/mL of the scaffold was used, the gap closure percentage increased with the amount of flaxseed extract added into the PVA nanofibers, both at 24 and 48 h. Specifically, at 24 h, gap closure percentages were 5.7% ± 0.5% (PVA), 37.4% ± 3.2% (P90/E10), 52.2% ± 3.0% (P80/E20), and 56.0% ± 4.6% (P70/E30). The extract effect was more obvious at 48 h: 67.0% ± 3.2% (PVA), 72.4% ± 3.5% (P90/E10), 82.0% ± 5.3% (P80/E20), and 84.1% ± 2.8% (P70/E30) (Figures 5A, 6A). At the concentration of 1 mg/mL, a similar pattern was observed. At 24 h, the gap closure percentages were 34.8% ± 1.5% (PVA), 32.8% ± 2.3% (P90/E10), 54.4% ± 2.0% (P80/E20), and 57.0% ± 2.2% (P70/E30). At 48 h, the gap closure percentages continued to increase: 69.9% ± 2.8% (PVA), 74.7% ± 3.1% (P90/E10), 95.5% ± 4.5% (P80/E20), and 97.7% ± 2.4% (P70/E30) (Figures 5B, 6B).

The results suggest that flaxseed extract significantly enhances MG-63 cell migration on nanofiber scaffolds, which is crucial for tissue regeneration. This cell migration-promoting effect of the flaxseed extract might be attributed to the presence of anti-inflammatory phenolic compounds that promotes better cell-material interactions, and larger nanofiber diameters that reinforce mechanical strength. These combined effects create a supportive environment for cell migration, indicating potential advantages for tissue regeneration in bone engineering. It is worth mentioning here that, cell migration assays inherently assess both the migratory and proliferative capabilities of cells. If gap closure were solely due to cell migration, a gap (cell-free area) would remain behind the migrating cells. Thus, the observed gap closure is a result of the combined effects of cell migration and proliferation. To accurately differentiate between these processes, future studies will incorporate additional assays, such as proliferation inhibition tests. These tests will help to isolate and verify the specific contributions of cell migration, providing a clearer understanding of the mechanisms involved in healing and cell movement.

### 3.7 MG-63 cell adhesion assay via crystal violet

**FIGURE 7:**
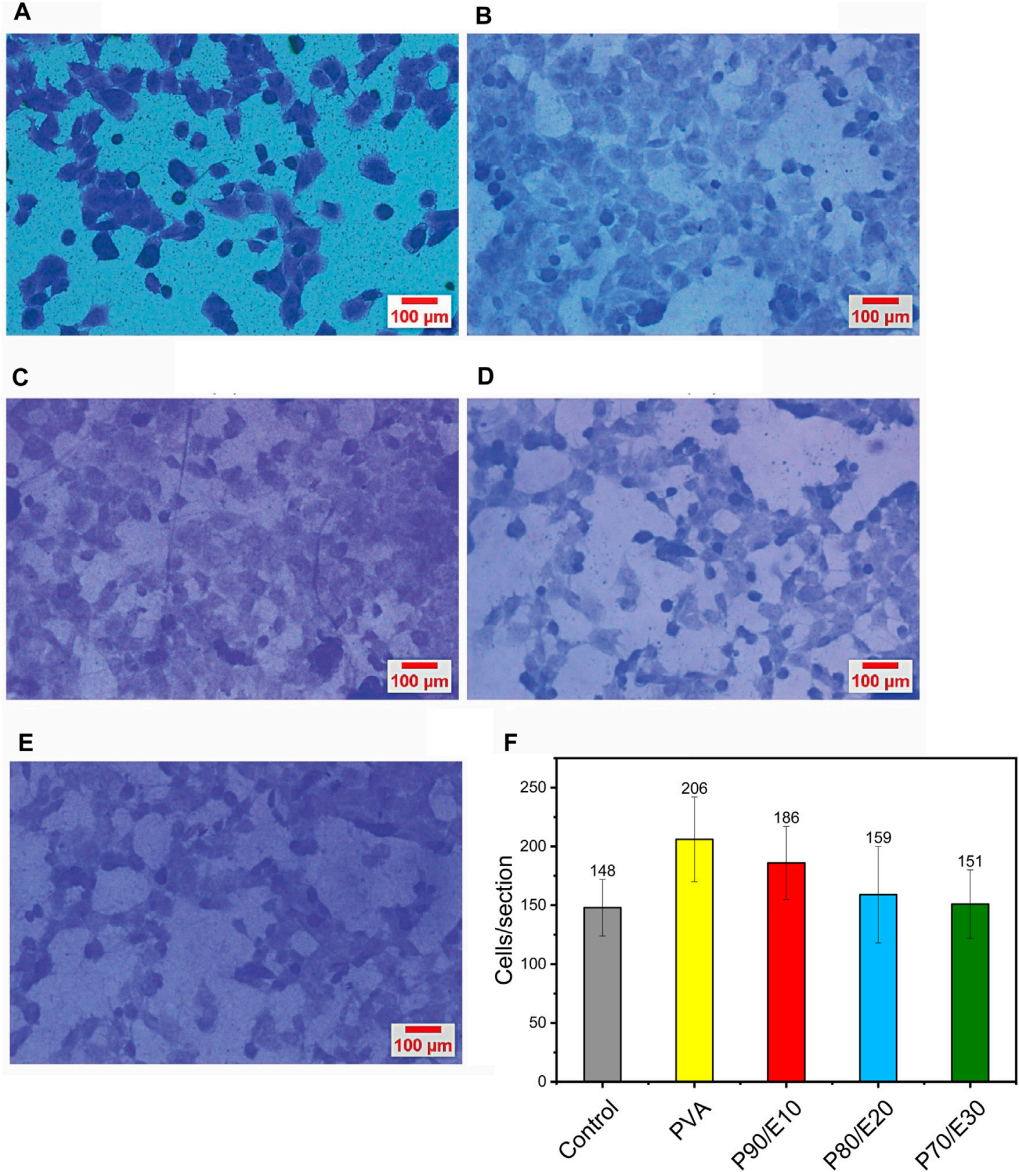
MG-63 cell adhesion evaluation by crystal violet staining after 2 h of incubation with **(A)** medium alone (control), **(B)** PVA, **(C)** P90/E10, **(D)** P80/E20, and **(E)** P70/E30 (0.5 mg/mL for all). **(F)** Quantification of adherent cells per section.

MG-63 cell adhesion (i.e., number of adherent cells per image section) was monitored with the crystal violet dye in the presence or not (control, medium alone) of the different scaffolds (Figure 7 and Supplementary Table S7). In the presence of 0.5 mg/mL scaffolds, the number of adherent cells per image section decreased with the flaxseed extract concentration. Specifically, after 48 h, the number of cells/section was 206 with PVA, 186 with P90/E10, 159 with P80/E20, and 151 with P70/E30. The slight (nonsignificant) reduction in cell adhesion, compared with pristine PVA NFs, is attributed to a minor decrease in cell hydrophilicity due to the flaxseed extract. Nevertheless, in the presence of nanofiber scaffolds with flaxseed extract, cells still exhibited cell adhesion values similar to those of control cells, emphasizing the scaffold’s overall biocompatibility and potential for cell attachment. These findings underscore the delicate balance required when designing nanofiber scaffolds for tissue engineering applications in which bioactive components (e.g., flaxseed extract) are incorporated while maintaining favorable surface properties for cell adhesion.

### 3.8 Osteogenic differentiation of MG-63 by quantitative real time polymerase chain reaction assay

**FIGURE 8:**
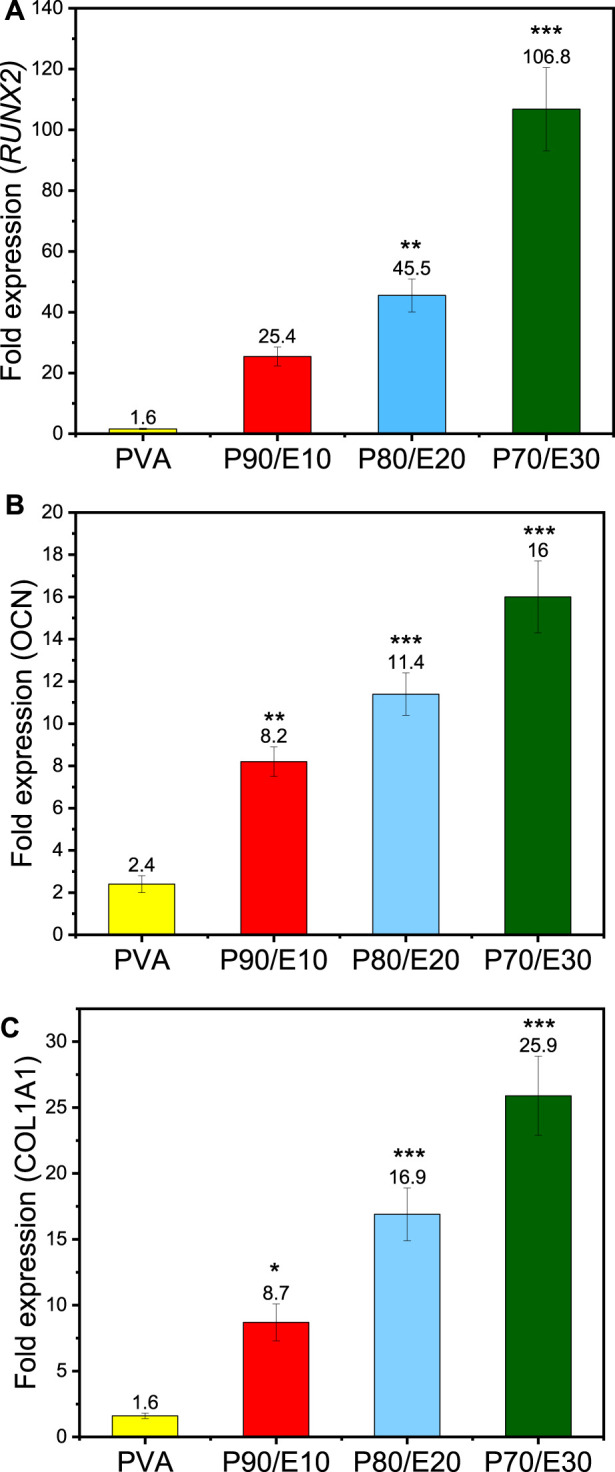
RT-qPCR analysis of MG-63 cells after incubation with the indicated nanofiber scaffolds at a concentration of 1 mg/mL for 3 days showing the fold expression in the expression of the **(A)** *RUNX2*, **(B)** *OCN*, and **(C)** *COL1A1* genes. One asterisk (*) indicates *p*-value < 0.05, two asterisks (**) indicate *p*-value < 0.01, and three asterisks (***) indicate *p*-value < 0.001 (compared to PVA NFs results).

Osteogenic differentiation of MG-63 cells cultured on PVA nanofiber scaffolds with varying concentrations of flaxseed extract was assessed by quantifying the expression of RUNX*2*, *COL1A1*, and *OCN* by RT-qPCR (Figure 8). Compared with control (medium alone), *RUNX2*, *COL1A1*, and *OCN* expression levels were increased by 1.6 ± 0.2-fold, 1.6 ± 0.2-fold, and 2.4 ± 0.4-fold in the presence of PVA and by 106.88 ± 13.75, 25.9 ± 3.1, and 16 ± 1.7 in the presence of P70/E30, respectively (all results in Supplementary Table S8). This significant upregulation indicates a robust promotion of osteogenic differentiation of MG-63 cells in the presence of the flaxseed extract. This positive effect on osteogenesis could be due to the high polyphenolic content in the extract. These findings highlight the positive impact of flaxseed extract-loaded nanofiber scaffolds on cellular activities related to bone regeneration, emphasizing their potential for bone tissue engineering applications. The effects of the fabricated nanofiber scaffolds on MG-63 cells, including cell viability, cell adhesion, cell migration, and cell osteogenesis, are summarized in Supplementary Table S9.

Osteogenesis promotion by polyphenols is explained at the molecular level by their effects on several osteogenic-related signaling pathways, including the estrogen receptor (ER) signaling pathway (Cauley, 2015), mitogen-activated protein kinases (MAPK) (Torre, 2017), Wnt/β-catenin (Torre, 2017), and tumor growth factor-beta/bone morphogenetic protein (TGF-β/BMP) (Melguizo-Rodríguez et al., 2019). Phytoestrogens, a class of polyphenols, can bind to estrogen receptors, modulating the expression of genes involved in bone mass maintenance (Cauley, 2015). It has been shown that osteogenic markers, such as *RUNX2* and *OCN*, are upregulated through ER signaling pathways (Xiao et al., 2014). The MAPK cascade, which regulates extracellular signal transduction, can be activated by polyphenols, leading to osteogenic effects (Torre, 2017). Moreover, polyphenols (e.g., genistein) induce MAPK phosphorylation, resulting in the upregulation of the *COL1A1*, *OCN* and *BMP6* genes that stimulate osteogenesis. Polyphenols also modulate the Wnt/β-catenin pathway, promoting osteogenesis (Liao et al., 2014). For instance, berry-derived polyphenols activate Wnt signaling pathways, leading to the activation of transcriptional factors, such as *RUNX2* and osterix (*OSX*), that promote osteoblastogenesis (Chen et al., 2014). Induction of the BMP signaling pathway by polyphenols enhances bone anabolism by upregulating the *BMP2* promoter and promoting osteogenesis. Silibinin, a polyphenolic compound, activates BMP signaling, leading to SMAD phosphorylation and upregulation of osteogenic markers, such as *RUNX2*, *ALP*, *OCN* and *COL1A1* (Ying et al., 2013). Our RT-qPCR results (Figure 8) further support the osteogenic potential of the fabricated nanofiber scaffolds, demonstrating the positive influence of flaxseed extract on osteogenic gene expression levels in MG-63 cells.

### 3.9 Future perspective and evaluation in the context of prior research

This study unveils the possibility of integrating flaxseed extracts into PVA nanofibers for bone tissue engineering. Nevertheless, several technical challenges and opportunities for refinement must be acknowledged. The slight reduction in cell adhesion attributed to the decreased hydrophilicity of the flaxseed extract-loaded scaffolds underscores the delicate balance required when incorporating bioactive components and the need of further optimization of the nanofiber surface properties. Additionally, the observed decrease of the swelling ratio with higher extract concentrations raises the question of potential alterations in the nanofiber scaffold structure over time. To address these challenges, the nanofiber scaffold formulation could be finely modified to enhance hydrophilicity without compromising other essential characteristics. Furthermore, although hydrolytic degradation was slowed down, future studies should monitor this parameter for longer periods to ensure sustained structural integrity. Recommendations for future research include exploring the long-term effects of the PVA/extract nanofiber scaffolds in an *in vivo* setting to provide a more comprehensive understanding of their regenerative potential. This study, through the meticulous control of essential parameters, comprehensive biological evaluation, and emphasis on gene expression analysis, provides a robust foundation for future advancements in flaxseed extract-loaded nanofibers for bone tissue engineering. The study findings encourage the continued exploration of these innovative materials, while highlighting the importance of refining the formulation to meet the complex demands of regenerative medicine. Table 3 summarizes the previously conducted studies utilizing the potential of incorporating plant extracts into polymeric nanofibers and their potential in bone tissue engineering applications.

**TABLE 3:** Summary of previous studies that used plant extracts to produce nanofiber scaffolds for bone tissue engineering applications.

Nanofiber composition	Plant extract	Morphology	Physical characteristics	Biological characteristic	References
Poly-ε-caprolactone (PCL)	*Wattakaka volubilis* active phytochemicals	Uniform bead-less, randomly oriented nanofibers with increased diameters following extract loading.	Loading of the extract resulted in a higher degradation rate compared with neat PCL nanofibers.	-MTT assay showed that the viability of primary human meniscus and osteoblast-like cells was increased in the presence of extract-loaded nanofibers compared with neat PCL nanofibers. -Hoechst staining showed higher DNA content in cells that adhered on extract-loaded nanofibers compared with neat PCL nanofibers. -Higher production of collagen and glycosaminoglycans was observed in cell cultures with extract-loaded nanofibers.	Venugopal et al. (2019)
Poly (L-lactic acid) (PLLA)	*Cissus quadrangularis* extract	Uniform bead-less, randomly oriented nanofibers with decreased diameters upon extract loading.	-Increased tensile strength upon extract loading (20 w/w%) -Reduced contact angle upon extract loading. -Higher protein adsorption on extract-loaded nanofibers due to higher hydrophilicity.	-Higher adhesion and distribution of mesenchymal stem cells on extract-loaded nanofibers due to their higher hydrophilicity. -Lactate dehydrogenase viability assay showed higher viability of cells cultured on extract-loaded nanofibers. -Higher alkaline phosphatase levels in cultures with extract-loaded nanofibers. -Alizarin Red staining showed higher mineralization levels in cultures with extract-loaded nanofibers.	Parvathi et al. (2018)
PCL-PEG-PCL	*Elaeagnus angustifolia* extract	Uniform bead-less randomly oriented nanofibers with increased diameters upon extract loading.	-Increase tensile strength upon extract loading. -Decreased contact angle upon extract loading.	-MTT assay showed higher viability of human dental pulp stem cells (hDPSCs) in the presence of extract-loaded nanofibers. -SEM images showed higher hDPSC adhesion on extract-loaded nanofibers. -Higher alkaline phosphatase activity in hDPSCs cultured on extract-loaded nanofibers. -RT-qPCR showed higher expression of *RUNX2*, *BMP2*, and *BGLAP* in hDPSCs seeded on extract- loaded nanofibers.	Hokmabad et al. (2019)
PVA	Flaxseed extract	Uniform bead-less, randomly oriented nanofibers with increased diameters upon extract loading.	Increased contact angle and tensile strength, decreased swelling ratio and hydrolytic degradation upon flaxseed extract loading.	-MTT assay showed higher MG-63 osteoblast viability upon extract loading. -Cell migration assay showed higher gap closure rates in cultures with extract-loaded scaffolds. -RT-qPCR showed higher upregulation of key osteogenic markers (*RUNX2*, *OCN* and *COL1A1*) with higher flaxseed extract concentrations.	This work

## 4 Conclusion

The integration of ethanolic flaxseed extracts into PVA nanofibers for bone tissue engineering applications has been explored through a comprehensive physical, biological, and molecular analysis. The meticulous control of essential parameters (fiber diameter, wettability, tensile strength, swelling ratio, and hydrolytic degradation) was crucial to optimize the nanofiber scaffolds for enhanced cellular responses. The nanofibrous scaffolds, particularly those loaded with flaxseed extract, displayed favorable characteristics, such as bead-free structures and varying fiber diameters (252.33 ± 62.03 nm for PVA and 435.03 ± 119.56 nm for P70/E30), thus addressing the requirements for successful bone tissue regeneration. The biological evaluation with MG-63 osteoblast-like cells showed enhanced cell proliferation and migration (MTT and cell migration assays) on flaxseed-loaded nanofibers. Despite the decrease in cell adhesion (206 ± 36 cells/section for PVA and 151 ± 29 cells/section for P70/E30) attributed to their reduced hydrophilicity, extract-loaded nanofibers promoted the differentiation of MG-63 cells, as indicated by the significant upregulation (RT-qPCR) of osteogenic markers (*RUNX2*, *COL1A1* and *OCN*). The improved hydrolytic degradation stability (100% of weight loss at day 2 for PVA vs. day 7 for P70/E30) further emphasizes the robustness due to the flaxseed extract incorporation. However, the swelling ratio data (1371% ± 127% for PVA and 702% ± 74% for P70/E30) indicated a notable decrease in water absorption capabilities. The MTT assay provided strong evidence of the nanofiber scaffold cytocompatibility and their potential to support cell viability. Overall, these findings underscore the potential of plant extract-loaded nanofibers, particularly flaxseed extracts, as advanced materials for bone tissue engineering that can promote osteogenic differentiation and cell functions crucial for successful tissue regeneration.

## Data Availability

The original contributions presented in the study are included in the article/Supplementary Material, further inquiries can be directed to the corresponding author.
