# Supplementary material for: Development of polyvinyl alcohol nanofiber scaffolds loaded with flaxseed extract for bone regeneration: phytochemicals, cell proliferation, adhesion, and osteogenic gene expression
Source: Front Chem. 2024 Jul 31;12:1417407. doi: 10.3389/fchem.2024.1417407 (PMC11322085; doi:10.3389/fchem.2024.1417407)
Supplement: Supplementary file 1 [file DataSheet1.docx]

**Development of Polyvinyl Alcohol Nanofiber Scaffolds Loaded with Flaxseed Extract for Bone Regeneration: Phytochemicals, Cell Proliferation, Adhesion, and Osteogenic Gene Expression**

Ahmed G. Abdelaziz,^1^ Hassan Nageh,^2^ Mohga S. Abdalla,^1^ Sara M. Abdo^1^, Asmaa A. Amer,^3^ Samah A. Loutfy,^2,4^ Nasra F Abdel Fattah,^4^ Ali Alsalme,^5^ David Cornu~~,~~^~~6~~^ Mikhael Bechelany,^6,7^ Ahmed Barhoum^8^

^1^ Biochemistry Division, Chemistry Department, Faculty of Science, Helwan University, Cairo, Egypt, [Ahmed.G.Abdelaziz@science.helwan.edu.eg](mailto:Ahmed.G.Abdelaziz@science.helwan.edu.eg), [Mohga.shafiek@science.helwan.edu.eg](mailto:Mohga.shafiek@science.helwan.edu.eg), [Sara.Mohamed@science.helwan.edu.eg](mailto:Sara.Mohamed@science.helwan.edu.eg)

^2^ Nanotechnology Research Centre (NTRC), The British University in Egypt, El-Shorouk City, Suez Desert Road, P.O. Box 43, Cairo 11837, Egypt, [Hassan.Nageh@bue.edu.eg](mailto:Hassan.Nageh@bue.edu.eg), [samah.loutfy@bue.edu.eg](mailto:samah.loutfy@bue.edu.eg)

^3^ Department of Pharmacognosy, Pharmaceutical and Drug Industries Research Institute, National Research Centre, Dokki, 12622, Cairo, Egypt, [Asmaa_3amer86@hotmail.com](mailto:Asmaa_3amer86@hotmail.com)

^4^ Virology and Immunology unit, Cancer Biology Department, National Cancer Institute, Cairo University, Cairo, Egypt, [samah.loutfy@gmail.com](mailto:samah.loutfy@gmail.com), [nasra.fathy@nci.cu.edu.eg](mailto:nasra.fathy@nci.cu.edu.eg)

^5^ Department of Chemistry, College of Science, King Saud University, Riyadh, 11451, Saudi Arabia

^6^ Institut Européen des Membranes (IEM), UMR 5635, University of Montpellier, ENSCM, CNRS, Montpellier, France

^7^ Gulf University for Science and Technology, GUST, [Masjid Al Aqsa Street, Mubarak Al-Abdullah, Kuwait](https://www.google.com/maps/place/data=!4m2!3m1!1s0x3fcf9e4faba8f719:0x9f258810391745be?sa=X&ved=2ahUKEwi15_CSn-iEAxUdWkEAHQbPBoIQ4kB6BAg0EAA)

^8^ NanoStruc Research Group, Chemistry Department, Faculty of Science, Helwan University, Cairo, Egypt.

***Chromatographic analysis of the flaxseed ethanolic extract (FEE)***

FEE components were identified by high performance liquid chromatography (HPLC, Aiglent Technologies, Agilent 1260 series, Waldbronne, Germany). The Eclipse C18 column (4.6 mm × 250 mm i.d., 5 μm) was used for the separation process. The mobile phase included deionized milli-Q water and 0.05% trifluoroacetic acid dissolved in acetonitrile. The separation program was designed to mix the mobile phase components as follows: 82% water at 0 min, 80% water from 0 to 5 min, 60% water from 5 to 12 min, 82% water from 12 to 15 min, and 85% water from 15 to 16 min. Phenolic compounds were identified based on their UV spectra and their retention times within the chromatographic column. About 10 µL of FEE dissolved in the mobile phase was injected. The detector wavelength was set at 280 nm and the temperature at 35 °C.

***Separation and identification of compounds in the FEE***

The PuriFlash 4100 system, with the Interchim software 5.0 (Interchim; Montluçon, France) and a PDA-UV-Vis detector 190-840 nm, was used for the preparative separations of the FEE (1 g) components*.* The separation was carried out using a C_18_-HP column (30 µm). The mobile phase consisting of 1% formic acid (A) and acetonitrile (B) was programmed for a gradient elution. Fifty fractions were obtained and were inspected by paper chromatography 1MM (CPP IMM) with butanol: acetic acid: water (4:1:5) and 15% acetic acid as running system. Similar fractions were combined and resulted in seven sub-fractions (A–G). These sub-fractions were further purified using a Sephadex LH-20 column and eluents with different polarities. 10 compounds were isolated, structurally elucidated and identified based on their physical, chemical, chromatographic and spectral data (UV, NMR, and MS) [6–8].

**
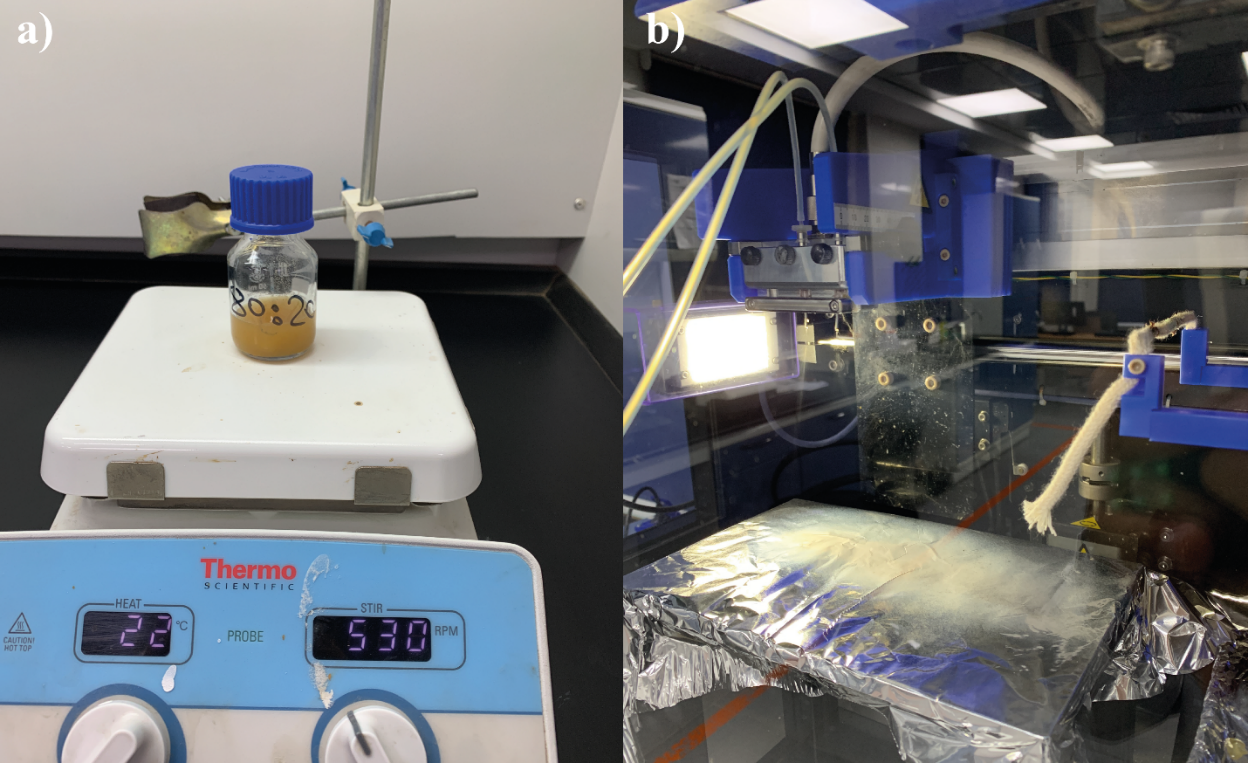
**

Figure S1: (a) Preparation of the electrospinning solutions. (b) Nanofiber fabrication by electrospinning.

**Table S1**: Electrospinning solution composition and processing parameters.

| Nanofiber scaffolds | Electrospinning solution composition and processing parameters | | | | | | |
| --- | --- | --- | --- | --- | --- | --- | --- |
|  | PVA  (g) | Flaxseed extract  (g) | Citric acid  (g) | Water  (mL) | Applied voltage (kV) | Flow rate (mL/h) | Tip to collector distance (cm) |
| PVA | 1.275 | 0 | 0.225 | 13.5 | 28 | 1.2 | 12.5 |
| P90/E10 | 1.125 | 0.15 | 0.225 | 13.5 | 29 | 1.1 | 12.5 |
| P80/E20 | 0.975 | 0.3 | 0.225 | 13.5 | 29.5 | 0.9 | 12.5 |
| P70/E30 | 0.825 | 0.45 | 0.225 | 13.5 | 30 | 0.7 | 12.5 |

**Table S2:** Physical characteristics of the prepared nanofiber scaffolds.

| Nanofiber  scaffolds | Physical characteristics | | | |
| --- | --- | --- | --- | --- |
|  | Fiber diameter  (nm) | Water contact angle (◦) | Weight loss upon immersion in water (%) | Swelling ratio after 48 h (%) |
| PVA | 252 ± 62 | 26.0 ± 1.6 | 100% after 2 days | 1371 ± 127 |
| P90/E10 | 276 ± 124 | 34.8 ± 0.9 | 100% after 6 days | 1102 ± 107 |
| P80/E20 | 326 ± 104 | 38.0 ± 1.4 | 100% after 6 days | 831 ± 89 |
| P70/E30 | 435 ± 119 | 45.9 ± 0.6 | 100% after 7 days | 702 ± 74 |

**Table S3:** Young’s modulus, elongation at break (%), and contact angle of the fabricated nanofibers.

| Sample | Young’s modulus (MPa) | Elongation at break (%) | Contact angle  (θ) |
| --- | --- | --- | --- |
| PVA | 310 ± 41 | 13.2 ± 1.3 | 25.9 ± 1.3 |
| P90/E10 | 1114 ± 127 | 9 ± 1 | 34.8 ± 0.9 |
| P80/E20 | 1292 ± 159 | 5 ± 0.4 | 37.9 ± 1.3 |
| P70/E30 | 4167 ± 391 | 3 ± 0.3 | 45.9 ± 0.6 |

**Table S4:** Hydrolytic degradation and swelling ratio of the fabricated nanofibers.

| Sample | Hydrolytic degradation (weight loss %) | | | | | | | Swelling ratio (%) | | | |
| --- | --- | --- | --- | --- | --- | --- | --- | --- | --- | --- | --- |
|  | Day 1 | Day 2 | Day 3 | Day  4 | Day 5 | Day  6 | Day  7 | 12 h | 24 h | 36 h | 48 h |
| PVA | 64.3 ± 2.6 | 100.0  ± 4.2 | - | - | - | - | - | 821 ± 71 | 904 ± 86 | 1130 ±105 | 1371 ±127 |
| P90/E10 | 43.5 ± 1.4 | 68.8 ± 3.5 | 81.6  ±4.1 | 89.1 ±6.0 | 93.1 ±5.5 | 100.0  ±7.3 | - | 713 ±64 | 781 ±83 | 906 ±103 | 1102 ±107 |
| P80/E20 | 35.6 ± 2.6 | 54.7 ± 3.1 | 67.8 ±5.2 | 82.0 ±7.5 | 91.5 ±8.3 | 100.0 ±9.8 | - | 593 ±61 | 664 ±78 | 738 ±84 | 831 ±89 |
| P70/E30 | 28.9 ± 2.1 | 44 ± 3.8 | 61.5 ±5.1 | 79.1 ±5.1 | 84.9 ±5.3 | 92.4 ±7.3 | 100 ±8 | 421 ±39 | 554 ±46 | 591 ±61 | 702 ±74 |

**Table S5:** MG-63 cell viability (%; MTT assay) after incubation with or without (control) flaxseed extract (E), PVA or extract-loaded PVA nanofibers at the indicated concentrations for 24 h.

| Condition | Cell viability (%) |
| --- | --- |
| Control (medium alone) | 100 ± 9.1 |
| E 25 µg/mL | 77.2 ± 9.6 |
| E 50 µg/mL | 84.6 ± 5.7 |
| E 100 µg/mL | 86.4 ± 6.5 |
| E 200 µg/mL | 93.9 ± 5.9 |
| E 400 µg/mL | 108 ± 7.1 |
| PVA 0.5 mg/mL | 72.2 ± 8.8 |
| PVA 1 mg/mL | 74.5 ± 6.8 |
| P90/E10 0.5 mg/mL | 78 ± 6.5 |
| P90/E10 1 mg/mL | 85.9 ± 7.9 |
| P80/E20 0.5 mg/mL | 90.1 ± 5.2 |
| P80/E20 1 mg/mL | 97.1 ± 7.2 |
| P70/E30 0.5 mg/mL | 95.6 ± 5 |
| P70/E30 1 mg/mL | 102.6 ± 8.8 |

**Table S6:** Wound closure (%) in MG-63 cell cultures in the presence or not (control) of the indicated scaffolds.

| Condition | Wound closure (%) | |
| --- | --- | --- |
|  | 24 h | 48 h |
| Control | 42 ± 2.6 | 58.6 ± 3.5 |
| PVA 0.5 mg/ml | 5.7 ± 0.5 | 67 ± 3.2 |
| PVA 1 mg/mL | 34.8 ± 1.5 | 69.9 ± 2.8 |
| P90/E10 0.5 mg/mL | 37.4 ± 3.2 | 72.4 ± 3.5 |
| P90/E10 1 mg/mL | 32.8 ± 2.3 | 74.7 ± 3.1 |
| P80/E20 0.5 mg/mL | 52.2 ± 3 | 82 ± 5.3 |
| P80/E20 1 mg/mL | 54.4 ± 2 | 95.5 ± 4.5 |
| P70/E30 0.5 mg/mL | 56 ± 4.6 | 84.1 ± 2.8 |
| P70/E30 1 mg/mL | 57 ± 2.2 | 97.7 ± 2.4 |

**Table S7:** Number of adherent MG-63 cells/section in the different conditions after crystal violet staining.

| Condition | Number of adherent cells/section |
| --- | --- |
| Control (medium alone) | 148 ± 24 |
| PVA | 206 ± 36 |
| P90/E10 | 186 ± 31 |
| P80/E20 | 159 ± 41 |
| P70/E30 | 151 ± 29 |

**Table S8:** Fold change of *RUNX2*, *OCN* and *COL1A1* gene expression (RT-qPCR) in MG-63 cells incubated with the indicated scaffolds relative to control (medium alone).

| Condition | Fold change | | |
| --- | --- | --- | --- |
|  | *RUNX2* | *OCN* | *COL1A1* |
| PVA | 1.6 ± 0.2 | 2.4 ± 0.4 | 1.6 ± 0.2 |
| P90/E10 | 25.4 ± 3.1 | 8.2 ± 0.7 | 8.7 ± 1.4 |
| P80/E20 | 45.5 ± 5.4 | 11.4 ± 1 | 16.9 ± 2 |
| P70/E30 | 106.8 ± 13.7 | 16 ± 1.7 | 25.9 ± 3 |

**Table S9:** Biological properties of the different nanofiber scaffolds (pristine PVA nanofibers vs PVA nanofibers loaded with the flaxseed extract).

| Nanofiber scaffolds | MTT assay –  Cell viability (%) | | Cell migration assay –  Wound closure (%) | | | | RT-qPCR assay – Fold  change | | | Cell adhesion assay  – N. of adherent cells/section |
| --- | --- | --- | --- | --- | --- | --- | --- | --- | --- | --- |
|  | 0.5 mg/ml | 1.0 mg/ml | 24 h | | 48 h | | *RUNX2* | *OCN* | *COL1A1* |  |
|  |  |  | 0.5 mg/ml | 1.0 mg/ml | 0.5 mg/ml | 1.0 mg/ml |  |  |  |  |
| PVA | 72.2  ± 8.8 | 74.5  ± 6.8 | 5.7 ± 2.6 | 34.8 ± 1.5 | 67 ± 3.2 | 69.9 ± 2.8 | 1.6 ± 0.2 | 2.4 ± 0.2 | 1.6 ± 0.4 | 206 ± 36 |
| P90/E10 | 78 ± 6.5 | 85.9 ± 7.9 | 37.4 ± 3.2 | 32.8 ± 2.3 | 72.4 ±3.5 | 74.5 ± 3.1 | 25.4 ± 3.1 | 8.2 ± 0.7 | 8.7 ± 1.4 | 186 ± 31 |
| P80/E20 | 90.1 ± 5.2 | 97.1 ± 7.2 | 52.2 ± 3.0 | 54.4 ± 2.0 | 82.0 ±5.3 | 95.5 ± 4.5 | 45.5 ± 5.1 | 11.4 ± 1.0 | 16.9 ± 2.0 | 159 ± 41 |
| P70/E30 | 95.6 ± 5.0 | 102.6 ± 8.8 | 56.0 ± 4.6 | 57.0 ± 2.3 | 4.1 ±2.8 | 97.7 ± 2.4 | 106.8 ±13.7 | 16.0 ± 1.7 | 25.9 ± 3.0 | 151 ± 29 |

**References:**

[1] T. Álvarez, R. Ramírez, In vitro Antimicrobial Activity and Phytochemical Analysis of Some Indian Medicinal Plants, Turkish Journal of Biology 31 (2007) 53–58. https://doi.org/-.

[2] K. Prabha, P. Karar, S. Hemalatha, A COMPARATIVE PRELIMINARY PHYTOCHEMICAL SCREENING ON THE LEAVES, STEM AND ROOTS OF THREE VIBURNUM LINN SPECIES, (2011).

[3] K. Prabha, P. Karar, S. Hemalatha, A COMPARATIVE PRELIMINARY PHYTOCHEMICAL SCREENING ON THE LEAVES, STEM AND ROOTS OF THREE VIBURNUM LINN SPECIES, Der Pharm. Sin 2 (2011) 81–93.

[4] A.A. Amer, R.S. Mohammed, Y. Hussein, A.S.M. Ali, A.A. Khalil, Development of Lepidium sativum Extracts/PVA Electrospun Nanofibers as Wound Healing Dressing, ACS Omega 7 (2022) 20683–20695. https://doi.org/10.1021/ACSOMEGA.2C00912/ASSET/IMAGES/LARGE/AO2C00912_0006.JPEG.

[5] E. Attard, A rapid microtitre plate Folin-Ciocalteu method for the assessment of polyphenols, Open Life Sci 8 (2013) 48–53.

[6] P.K. Agrawal, Flavonoids, Carbon-13 NMR of Flavonoids 564 (1989).

[7] K.R. Markham, others, Techniques of flavonoid identification., Academic press, 1982.

[8] T. Mabry, K.R. Markham, M.B. Thomas, The systematic identification of flavonoids, Springer Science \& Business Media, 2012.
